# Alcohol consumption and fecundability: prospective Danish cohort study

**DOI:** 10.1136/bmj.i4262

**Published:** 2016-08-31

**Authors:** Ellen M Mikkelsen, Anders H Riis, Lauren A Wise, Elizabeth E Hatch, Kenneth J Rothman, Heidi T Cueto, Henrik Toft Sørensen

**Affiliations:** 1 Department of Clinical Epidemiology, Aarhus University Hospital, 8200 Aarhus N, Denmark; 2 Department of Epidemiology, Boston University School of Public Health, Boston, MA, 617857, USA; 3 RTI Health Solutions, Research Triangle Park, NC, 27709 USA

## Abstract

**Objective** To investigate to what extent alcohol consumption affects female fecundability.

**Design** Prospective cohort study.

**Setting** Denmark, 1 June 2007 to 5 January 2016.

**Participants** 6120 female Danish residents, aged 21-45 years, in a stable relationship with a male partner, who were trying to conceive and not receiving fertility treatment.

**Main outcome measures** Alcohol consumption was self reported as beer (330 mL bottles), red or white wine (120 mL glasses), dessert wine (50 mL glasses), and spirits (20 mL) and categorized in standard servings per week (none, 1-3, 4-7, 8-13, and ≥14). Participants contributed menstrual cycles at risk until the report of pregnancy, start of fertility treatment, loss to follow-up, or end of observation (maximum 12 menstrual cycles). A proportional probability regression model was used to estimate fecundability ratios (cycle specific probability of conception among exposed women divided by that among unexposed women).

**Results** 4210 (69%) participants achieved a pregnancy during follow-up. Median alcohol intake was 2.0 (interquartile range 0-3.5) servings per week. Compared with no alcohol consumption, the adjusted fecundability ratios for alcohol consumption of 1-3, 4-7, 8-13, and 14 or more servings per week were 0.97 (95% confidence interval 0.91 to 1.03), 1.01 (0.93 to 1.10), 1.01 (0.87 to 1.16) and 0.82 (0.60 to 1.12), respectively. Compared with no alcohol intake, the adjusted fecundability ratios for women who consumed only wine (≥3 servings), beer (≥3 servings), or spirits (≥2 servings) were 1.05 (0.91 to1.21), 0.92 (0.65 to 1.29), and 0.85 (0.61 to 1.17), respectively. The data did not distinguish between regular and binge drinking, which may be important if large amounts of alcohol are consumed during the fertile window.

**Conclusion** Consumption of less than 14 servings of alcohol per week seemed to have no discernible effect on fertility. No appreciable difference in fecundability was observed by level of consumption of beer and wine.

## Introduction

For many women of reproductive age, alcohol consumption is an integral part of their lifestyle. In Denmark, more than 30% of women aged 16-34 years have a weekly intake of seven drinks or more, and 18.2% of American women aged 18-44 years engage in binge drinking (at least four drinks per episode) on average 3.2 times within 30 days.^1^ ^2^ ^3^ Alcohol consumption of more than one drink a day during pregnancy has been associated with low birth weight, preterm birth, and fetal alcohol spectrum disorders.^4^ ^5^ Official guidelines in several countries recommend maximum alcohol intake of seven drinks a week for non-pregnant women in general and no alcohol intake for pregnant women and women trying to conceive.^6^ ^7^ ^8^ Nonetheless, the extent to which alcohol consumption affects female fertility is unclear. Some studies have reported that low to moderate levels of alcohol consumption are associated with decreased fertility.^9^ ^10^ ^11^ Others found either no association^12^ ^13^ ^14^ ^15^ ^16^ ^17^ ^18^ or a positive association between moderate alcohol intake and fertility for men and women.^19^ ^20^ Wine may contain some healthful compounds, possibly accounting for the association between moderate consumption and beneficial effects on cardiovascular disease, diabetes, and osteoporosis.^21^ Few studies have evaluated the relation between specific types of alcohol and fertility. One study of 29 844 pregnant women identified from the Danish National Birth Cohort found that wine drinkers had a shorter time to pregnancy than women who did not drink wine.^16^ Most studies have examined pregnant women or women seeking fertility treatment and have collected retrospective data on alcohol consumption during the preconception period.^9^ ^13^ ^14^ ^15^ ^16^ ^17^ ^18^ ^19^ ^20^ These studies are susceptible to a variety of biases, including differential exposure misclassification (recall bias), left truncation bias, and selection bias.^22^ ^23^

In developed countries, up to 24% of couples experience infertility defined as time to pregnancy of 12 months or more,^24^ ^25^ ^26^ and alcohol consumption is considerable.^3^ Thus, the effect of alcohol consumption, especially at moderate levels (from one to seven servings a week), on fecundability is an important public health concern. Accordingly, in a prospective cohort of Danish women trying to conceive, we examined the association between preconception alcohol consumption and time to pregnancy, examining overall alcohol consumption as well as consumption of specific types of alcoholic beverage (beer, wine, and spirits).

## Methods

Data used in this study were collected as part of the prospective cohort study “SnartGravid” (Soon Pregnant) and its successor study “SnartForaeldre” (Soon Parents).^27^ ^28^ Enrollment and data collection procedures have been described in detail elsewhere.^28^ The study was publicized by online and offline media. Briefly, enrollment and data collection were managed via email and the study websites (www.Snart-gravid.dk (closed) and www.SnartForaeldre.dk). Potential participants volunteered for the study by accessing the study website, where they were required to read a consent form and complete a screener questionnaire to confirm eligibility. Eligible women were invited to complete a baseline questionnaire and bimonthly questionnaires for 12 months or until conception occurred, whichever occurred earlier. Baseline questions recorded data on sociodemographic background, medical and reproductive history, and behavioral and lifestyle factors. Follow-up questionnaires ascertained information on pregnancy status, date of last menstrual period, and lifestyle exposures likely to change over time such as alcohol use, frequency of intercourse, and smoking.

### Study population

We enrolled women who met the following criteria: aged 18-40 years, Danish resident, in a stable relationship with a male partner, attempting to conceive, and not receiving fertility treatment. In total, 9497 eligible women enrolled in the study from 1 June 2007 to 5 January 2016. This analysis excluded 1495 women who had tried to conceive for more than six months at study entry, 1139 women who did not complete at least one follow-up questionnaire, 426 women who reported incomplete or implausible information about their last menstrual period or date of first pregnancy attempt, 311 women who enrolled more than once, and six women who withdrew their consent. Thus, the final study population comprised 6120 women with at least eight weeks of follow-up.

### Assessment of alcohol exposure

We assessed alcohol consumption at baseline and at the time of each follow-up questionnaire by asking respondents to consider their alcohol intake during the previous month and to report their average weekly consumption of bottles of beer (330 mL) and glasses of red wine (120 mL), white wine (120 mL), dessert wine (50 mL), and spirits (20 mL). Help buttons in the web based questionnaire provided information on serving sizes in milliliters and instructed respondents who typically drank less than one serving a week to report “no intake.” We calculated total alcohol intake by summing the number of standard servings consumed across beverage types. We also categorized weekly alcohol intake in servings as none, one to three, four to seven, eight to 13, and 14 or more. For analyses based on type of alcoholic beverage, we classified weekly consumption as none, one, two, and three or more for wine and beer and as none, one, and two or more for spirits. We updated alcohol consumption for each menstrual cycle by using data from the most recent follow-up questionnaire before a study event was reported.

### Assessment of pregnancy and cycles at risk

The study endpoint was the occurrence of a pregnancy, regardless of its outcome. Pregnancy was confirmed with a home pregnancy test by 92% (3878/4210) of the participants who became pregnant. We measured time to pregnancy in cycles and estimated it as the number of days a woman had tried to conceive divided by the estimated cycle length. This interval includes both time before study entry, limited to a maximum of six months, and the time that each participant was followed during the study. Total cycles at risk were calculated as: (days of trying to achieve pregnancy at study entry/cycle length)+(((last menstrual period date from most recent follow-up questionnaire−date of baseline questionnaire completion)/cycle length)+1).^29^

### Assessment of covariates

The questionnaires provided data on age, partner’s age, parity, gravidity, vocational training, cycle regularity, height, weight, physical activity, last method of contraception, frequency of intercourse, timing of intercourse, sexually transmitted infections, smoking, and caffeine consumption. We used baseline data on weight, height, physical activity, and smoking history to calculate body mass index, total metabolic equivalents, and pack years of smoking. We calculated body mass index as weight (kg)/height (m) squared. In a validation study comparing self reported height and weight with birth registry based data on height and weight, the correlation coefficients for both variables were 0.96.^30^ We estimated total metabolic equivalents by summing the metabolic equivalents reported for moderate and vigorous physical activity—that is, hours per week multiplied by 3.5 and hours per week multiplied by 7.0, respectively.^31^

### Data analysis

At baseline, the proportion of missing data ranged from 0.2% (9/6120) for weight to 6.8% (414/6120) for dessert wine. We used multiple imputation to impute missing values for all baseline covariates. To impute missing values for the time dependent variables (intercourse frequency, alcohol and caffeine consumption), we used multiple imputation and last observation carried forward; the two methods yielded similar results. Multiple imputation involved using covariate and outcome variables combined with random error to generate five imputed datasets and then combining the results across the imputed datasets.^32^ In a sensitivity analysis, we generated 100 imputed datasets and included imputation of missing follow-up data.

We used proportional probabilities regression models to compute fecundability ratios and 95% confidence intervals.^33^ The fecundability ratio represents the cycle specific probability of conception among exposed women divided by that among unexposed women. A fecundability ratio below one indicates reduced fertility for exposed women compared with unexposed women.^33^ At enrollment, participants had been trying to conceive for a varying number of cycles, ranging from zero to six. We took into account left truncation of the data, basing compared risk sets on observed menstrual cycles at risk and preserving their ordinality relative to the start of pregnancy attempt time.^30^ Right censoring began when a participant started fertility treatment, stopped trying to conceive, stopped responding to the questionnaires, or reached the end of the observation period (12 cycles of pregnancy attempt).

In the multivariate regression analysis, we assessed the association between time to pregnancy and total alcohol consumption (standard servings) and for type of alcoholic beverages (beer, wine, and spirits). In the primary analyses, we modeled time varying alcohol as the main exposure; in secondary analyses, we modeled baseline exposure only. We used two regression models to control for possible confounding. In model 1, we adjusted for a large number of potential risk factors for subfertility (age, partner’s age, parity, vocational training, cycle regularity, body mass index, physical activity, last method of contraception, smoking, intercourse frequency, timing of intercourse, sexually transmitted infections, and caffeine intake). We chose these variables on the basis of the literature, clinical relevance, and their association with the exposure at baseline. Model 2 included woman’s age and variables (parity and timing of intercourse) that noticeably changed the fecundability ratio (≥3%). In addition, we repeated model 2 and adjusted for gravidity (0, 1, and ≥2) instead of parity (parous and nulliparous). Furthermore, we stratified the results according to parity (parous versus nulliparous), intercourse frequency (<4 versus ≥4 times/week), and timing of intercourse (yes versus no). Because women who have attempted pregnancy for several months may change behaviors, we repeated the analyses including only those women who had started their pregnancy attempts within two cycles before study entry. Finally, we used restricted cubic splines to allow a less restricted fit of the relation between total alcohol consumption and fecundability.^34^ We used SAS version 9.2 for all analyses.

### Patient involvement

No patients were involved in setting the research question or planning the study. Participants had the option to invite other women trying to conceive to visit the study website and join the study. All results are posted on the study website.

## Results

In total, 4210 (69%) of the 6120 participants achieved a pregnancy during follow-up. The median age of participants was 28 years at entry into the study, compared with 30 years for their male partners. More than two thirds of participants had attempted to become pregnant for two cycles or fewer at study entry. Overall, cohort retention was 88% (5379/6120). Relative to participants who completed the study, the 741 (12%) participants who were not under observation for the entire follow-up period were more likely to be nulliparous. However, the two groups were similar according to all other baseline characteristics, including alcohol consumption (data not shown). Overall, few data were missing and imputation using either five or 100 imputed datasets produced similar results.

At baseline, median alcohol intake was 2.0 (interquartile range 0-3.5) servings per week. More participants consumed wine (59%; 3591) than beer (38%; 2346) or spirits (24%; 1464). Of the 6210 participants, 2541 (41%) consumed a combination of at least two types of alcoholic beverage, and 1198 (20%), 311 (5%), and 222 (4%) participants consumed only wine, beer, or spirits, respectively. Older age of both partners, irregular menstrual cycles, physical activity, smoking, caffeine intake, history of sexually transmitted infections, lack of timing of intercourse, and short attempt time at study entry were associated with increased alcohol consumption at baseline (table 1). By contrast, being parous and having low education were inversely associated with alcohol consumption.

**Table 1 tbl1:** Baseline characteristics of 6120 participants by level of alcohol consumption. Values are numbers (percentages) unless stated otherwise

Characteristic	Alcohol intake in servings per week				
	None	1-3	4-7	8-13	≥14
No of women	1848	2801	1120	276	75
Median (IQR) age, years	27.0 (25.0-31.0)	28.0 (26.0-31.0)	29.0 (26.0-32.0)	29.0 (26.0-31.0)	29.0 (26.0-33.0)
Median (IQR) partner’s age, years	30.0 (27.0-33.0)	30.0 (27.0-34.0)	30.0 (28.0-34.0)	31.0 (28.0-33.0)	32.0 (29.0-35.0)
Irregular cycles	488 (26.4)	713 (25.5)	264 (23.6)	74 (27)	25 (33)
Median (IQR) cycle length, days	29.0 (28.0-31.0)	29.0 (28.0-32.0)	29.0 (28.0-31.0)	29.0 (28.0-31.0)	28.0 (28.0-31.0)
Parous, ever had live birth	705 (38.2)	955 (34.1)	319 (28.5)	59 (21)	17 (23)
Median (IQR) body mass index	23.2 (20.9-27.2)	22.9 (20.8-25.8)	22.6 (20.8-25.1)	22.8 (21.1-25.0)	22.2 (20.8-26.2)
Median (IQR) physical activity, MET hrs/week	29.5 (16.0-48.0)	29.7 (16.0-48.0)	32.0 (16.0-48.0)	31.9 (16.0-48.0)	32.0 (16.0-56.0)
Short vocational training (<3 years)	803 (43.5)	953 (34.0)	344 (30.7)	71 (26)	24 (32)
Current smoking	214 (11.6)	402 (14.4)	249 (22.2)	85 (31)	25 (33)
Caffeine consumption ≥150 g/day	417 (22.6)	1014 (36.2)	556 (49.6)	141 (51)	44 (59)
Mean (SD) pack years of smoking	1.5 (3.6)	1.6 (3.5)	1.9 (3.6)	2.3 (3.9)	4.5 (6.5)
Frequency of intercourse ≥4 times/week	376 (20.4)	502 (17.9)	194 (17.3)	63 (23)	15 (20)
No timing of intercourse	737 (39.9)	1232 (44.0)	561 (50.1)	149 (54)	40 (53)
Attempt time before study entry:					
0-1 cycles	960 (52.0)	1478 (52.8)	643 (57.4)	160 (58)	45 (60)
2-3 cycles	519 (28.1)	746 (26.6)	272 (24.3)	66 (24)	17 (23)
4-6 cycles	369 (20.0)	577 (20.6)	205 (18.3)	50 (18)	13 (17)
History of sexually transmitted infection	557 (30.1)	876 (31.3)	394 (35.2)	94 (34)	27 (36)

IQR=interquartile range; MET=total metabolic equivalents.

Compared with no alcohol consumption, the adjusted fecundability ratios for consumption of one to three, four to seven, eight to 13, and 14 or more servings of alcohol a week were 0.97 (95% confidence interval 0.91 to1.03), 1.01 (0.93 to 1.10), 1.01 (0.87 to 1.16), and 0.82 (0.60 to 1.12), respectively (table 2, model 2). Adjustment for gravidity as opposed to parity had little effect on the fecundability ratios (data not shown). In addition, the fecundability ratios were broadly similar when we used baseline exposure data instead of time varying exposure data (data not shown) and when we restricted the analysis to women who had attempted pregnancy for two or fewer cycles at study entry (fecundability ratio 1.17 (0.23 to 1.99) for 8-13 servings/week and 0.68 (0.23 to 1.99) for ≥14 servings/week).

**Table 2 tbl2:** Fecundability by amount of alcohol consumed per week and alcohol type (n=6120)

Alcohol servings/week	Pregnancies	Cycles	Fecundability ratio* (95% CI)		
			Unadjusted model	Adjusted model 1†	Adjusted model 2‡
None	1381	8054	1.00 (reference)	1.00 (reference)	1.00 (reference)
Any alcohol:					
1-3	1875	11 272	0.95 (0.89 to 1.01)	0.94 (0.88 to 1.00)	0.97 (0.91 to 1.03)
4-7	738	4334	0.96 (0.88 to 1.04)	0.97 (0.89 to 1.05)	1.01 (0.93 to 1.10)
8-13	179	1097	0.92 (0.80 to 1.06)	0.96 (0.83 to 1.11)	1.01 (0.87 to 1.16)
≥14	37	307	0.73 (0.54 to 1.00)	0.82 (0.60 to 1.12)	0.82 (0.60 to 1.12)
Wine§ only:					
1	460	2515	1.06 (0.96 to 1.16)	1.02 (0.93 to 1.12)	1.05 (0.95 to 1.15)
2	199	1215	0.93 (0.80 to 1.07)	0.90 (0.78 to 1.04)	0.95 (0.82 to 1.09)
≥3	169	926	1.01 (0.88 to 1.17)	1.03 (0.89 to 1.19)	1.05 (0.91 to 1.21)
Beer only:					
1	137	878	0.92 (0.78 to 1.09)	0.91 (0.78 to 1.08)	0.93 (0.79 to 1.09)
2	45	286	0.92 (0.70 to 1.21)	0.95 (0.72 to 1.25)	0.96 (0.73 to 1.26)
≥3	28	197	0.86 (0.61 to 1.21)	0.93 (0.66 to 1.30)	0.92 (0.65 to 1.29)
Spirits only:					
1	111	762	0.86 (0.71 to 1.03)	0.89 (0.74 to 1.07)	0.88 (0.73 to 1.05)
≥2	32	222	0.83 (0.60 to 1.14)	0.87 (0.63 to 1.21)	0.85 (0.61 to 1.17)

*Cycle specific probability of conception, comparing exposed with unexposed women.

†Alcohol intake adjusted for woman’s and male partner’s age at baseline, vocational training, cycle regularity, parity, current smoking, intercourse frequency, timing of intercourse, body mass index, physical activity, sexually transmitted diseases, caffeine intake, and last method of contraception.

‡Alcohol intake adjusted for woman’s age, parity, and timing of intercourse.

§Including red and white wine.

The association between high alcohol consumption and lower fecundability varied by parity status and timing of intercourse (table 3). Among nulliparous women, the adjusted fecundability ratio was 0.76 (0.51 to 1.11) for consumption of 14 or more servings a week relative to none; among parous women, the fecundability ratio was 0.96 (0.58 to 1.59). Alcohol consumption of 14 or more servings a week was more strongly inversely associated with lower fecundability for women who did not time their intercourse. Similarly to the categorical analyses, the restricted cubic spline curve indicated little association between low amounts of alcohol intake and fecundability (fig 1). In contrast to the categorical analysis, the spline curve shows that the inverse association between alcohol consumption and fecundability may start at around 10 servings a week, but the width of the confidence interval lines widens above 10 servings a week. Compared with no alcohol intake, the adjusted fecundability ratios for women who consumed only wine (≥3 servings), beer (≥3 servings), or spirits (≥2 servings) were 1.05 (0.91 to1.21), 0.92 (0.65 to 1.29), and 0.85 (0.61 to 1.17), respectively (table 2, model 2).

**Table 3 tbl3:** Alcohol consumption and fecundability stratified by parity, intercourse frequency, and timing of intercourse (n=6120)

	Alcohol consumption, standard servings per week				
	None	1-3	4-7	8-13	≥14	
**Nulliparous**						
Pregnancies	807	1162	497	143	25	
Cycles	5369	7940	3216	912	242	
FR (95% CI)	Reference	0.96 (0.89 to 1.05)	0.99 (0.89 to 1.10)	1.02 (0.87 to 1.21)	0.72 (0.49 to 1.06)	
Adjusted FR* (95% CI)	Reference	0.93 (0.85 to 1.01)	0.95 (0.85 to 1.06)	0.98 (0.83 to 1.16)	0.74 (0.50 to 1.09)	
Adjusted FR† (95% CI)	Reference	0.96 (0.88 to 1.04)	1.00 (0.90 to 1.11)	1.04 (0.88 to 1.23)	0.76 (0.51 to 1.11)	
**Parous**						
Pregnancies	574	713	241	36	12	
Cycles	2685	3332	1118	185	65	
FR (95% CI)	Reference	0.97 (0.88 to 1.06)	0.98 (0.86 to 1.11)	0.82 (0.61 to 1.12)	0.91 (0.55 to 1.50)	
Adjusted FR* (95% CI)	Reference	0.96 (0.87 to 1.06)	0.99 (0.86 to 1.13)	0.86 (0.63 to 1.17)	1.03 (0.62 to 1.72)	
Adjusted FR† (95% CI)	Reference	0.98 (0.89 to 1.08)	1.03 (0.90 to 1.18)	0.89 (0.65 to 1.21)	0.96 (0.58 to 1.59)	
**Intercourse frequency <4 per week**						
Pregnancies	1105	1530	604	143	30	
Cycles	6570	9324	3618	853	238	
FR (95% CI)	Reference	0.95 (0.89 to 1.02)	0.95 (0.87 to1.05)	0.96 (0.81 to 1.13)	0.76 (0.54 to 1.07)	
Adjusted FR* (95% CI)	Reference	0.94 (0.88 to 1.01)	0.96 (0.87 to 1.06)	1.00 (0.84 to 1.18)	0.85 (0.60 to 1.20)	
Adjusted FR† (95% CI)	Reference	0.98 (0.91 to 1.05)	1.02 (0.93 to 1.12)	1.06 (0.90 to 1.25)	0.85 (0.59 to 1.21)	
**Intercourse frequency ≥4 per week**						
Pregnancies	276	345	134	36	7	
Cycles	1484	1948	716	244	69	
FR (95% CI)	Reference	0.96 (0.83 to 1.11)	1.00 (0.83 to 1.20)	0.80 (0.58 to 1.09)	0.65 (0.32 to 1.32)	
Adjusted FR* (95% CI)	Reference	0.95 (0.81 to1.10)	0.99 (0.82 to 1.21)	0.84 (0.61 to 1.16)	0.71 (0.35 to 1.42)	
Adjusted FR† (95% CI)	Reference	0.94 (0.82 to1.09)	1.00 (0.83 to 1.21)	0.81 (0.59 to 1.11)	0.69 (0.35 to 1.39)	
**No timing of intercourse**						
Pregnancies	573	809	356	97	18	
Cycles	3600	5276	2179	614	178	
FR (95% CI)	Reference	0.94 (0.85 to 1.04)	0.99 (0.88 to 1.12)	0.97 (0.79 to 1.18)	0.64 (0.41 to 1.00)	
Adjusted FR* (95% CI)	Reference	0.90 (0.81 to 0.99)	0.96 (0.85 to 1.09)	0.95 (0.78 to 1.17)	0.69 (0.44 to 1.08)	
Adjusted FR† (95% CI)	Reference	0.94 (0.85 to 1.04)	1.03 (0.91 to1.17)	1.02 (0.84 to 1.24)	0.69 (0.44 to 1.07)	
**Timing of intercourse**						
Pregnancies	808	1066	382	82	19	
Cycles	4454	5996	2155	483	129	
FR (95% CI)	Reference	0.98 (0.90 to 1.06)	0.96 (0.86 to 1.07)	0.93 (0.75 to 1.15)	0.91 (0.59 to 1.41)	
Adjusted FR* (95% CI)	Reference	0.98 (0.90 to 1.06)	0.97 (0.86 to 1.09)	0.97 (0.78 to 1.21)	0.98 (0.64 to 1.52)	
Adjusted FR† (95% CI)	Reference	0.99 (0.91 to 1.07)	0.99 (0.89 to 1.11)	1.00 (0.81 to 1.25)	0.99 (0.64 to 1.52)	

FR=fecundability ratio (cycle specific probability of conception, comparing exposed with unexposed women).

*Adjusted for woman’s and male partner’s age at baseline, vocational training, cycle regularity, parity, current smoking, intercourse frequency, timing of intercourse, body mass index, physical activity, sexually transmitted diseases, caffeine intake, and last method of contraception.

†Adjusted for adjusted for woman’s age, parity, and timing of intercourse (when applicable).

**Figure f1:**
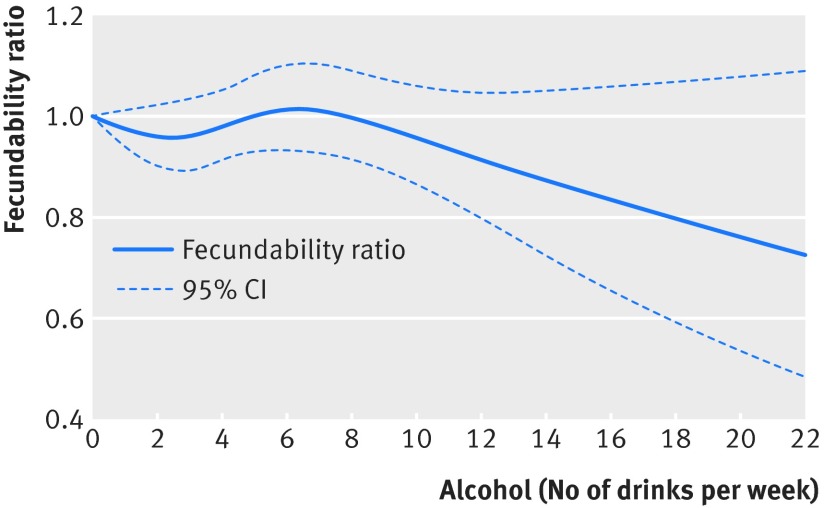
**Fig 1** Relation between amounts of alcohol consumed per week and fecundability, fitted by restricted cubic splines. Curves were adjusted for woman’s age, parity, and timing of intercourse (n=6120). Reference level is no alcohol consumed. Four knots were located at 0, 3, 5, and 10 drinks a week

## Discussion

In this prospective study of women trying to conceive, consumption of the highest amount of alcohol (≥14 servings a week) was associated with an 18% decrease in fecundability compared with no alcohol consumption, although the confidence interval of the estimate was wide. However, the results did not support an effect of alcohol consumption at more moderate levels (one to seven servings a week). We observed no appreciable differences in the associations of consumption of beer and wine with fecundability, whereas the association between consumption of spirits and fecundability was slightly stronger.

### Strengths and limitations of study

Drawn from the general population, our study population represents the full spectrum of fertility, including a mixture of highly fertile and less fertile women. The prospective design limits selection bias, as participants enrolled before pregnancy occurred. Study retention was high, and women with complete follow-up and partial follow-up had similar distributions of alcohol consumption and most other baseline characteristics. Of course, some differences likely exist between our study population and all Danish women, as well as women in other countries. These differences should not affect the validity of comparisons between categories of women within our study. In addition, a recent validation study comparing well established perinatal associations among members of our internet based cohort and all other Danish women giving birth indicated that selection bias was not a major concern for the associations selected for study.^35^

We adjusted for a large number of potential confounders, including timing and frequency of intercourse and last method of contraception. However, our data did not distinguish between regular drinking and binge drinking. This distinction may be important if large amounts of alcohol are consumed during the fertile window during which menstrual cycle disturbances related to alcohol are most prominent.^36^ If binge drinking during the fertile window was prevalent among the participants, we may have overestimated any association between regular alcohol consumption and fecundability. In addition, we lacked information on consumption of alcoholic beverages by male partners, which may be related both to the female partner’s alcohol consumption and to decreased sperm quality.^37^ ^38^ To reduce potential exposure misclassification, we provided information on serving sizes via “pop-up boxes” in the online questionnaire, and alcohol consumption was updated during the follow-up period. However, self reported alcohol intake was not validated. If alcohol intake is inaccurately reported, it seems more likely to be under-reported.^39^ If under-reporting is consistent across levels of intake, the ranking of effects by alcohol consumption is likely to be as reported, but the amounts of alcohol that correspond to the reported effects would be greater than reported.

### Comparison with other studies

Compared with other prospective studies,^10^ ^11^ ^19^ our study sample is relatively large; however, the number of women consuming 14 or more servings of alcohol a week and the number of women exclusively consuming one type of alcoholic beverage in amounts of more than two servings a week are small, so the estimates for these exposures are imprecise. Studies based on self reported retrospective time to pregnancy data and alcohol consumption found results similar to ours—namely, that high alcohol intake (>14 drinks/week) but not low or moderate intake was associated with decreased fecundability.^15^ ^18^ One prospective study of 259 couples, which compared weekly intake of less than five drinks with five to 10 and 10 or more drinks, found little association between alcohol intake and fecundability.^19^ In addition, in a recent nested case-control study of 686 case-control pairs in which alcohol consumption was assessed at baseline and the outcome “women reporting difficulty to get pregnant” was assessed biennially, no association was observed for any level of alcohol consumption.^12^ In contrast, two prospective studies by Jensen et al and Hakim et al both reported an association between low to moderate amounts of alcohol intake and fecundability.^10^ ^11^ Among 430 couples with no previous pregnancy, Jensen et al reported adjusted fecundability odds ratios for one to five, six to 10, 11 to 15, and more than 15 drinks a week of 0.61 (95% confidence interval 0.40 to 0.93), 0.55 (0.36 to 0.85), 0.34 (0.22 to 0.52), and 0.34 (0.11 to 1.07), compared with no alcohol intake.^11^ In a study of 124 women trying to conceive, Hakim et al and found that, compared with no intake of alcohol, drinking even less than one drink a week was associated with reduced fecundability. Their data, however, did not exhibit a consistent dose-response relation across levels of intake (0.43 (0.25 to 0.76), 0.40 (0.21 to 0.77), and 0.65 (0.20 to 2.15) for less than one drink, one to seven drinks, and more than seven drinks a week, respectively).^10^ The difference between the results of these two studies and our study may be explained in part by differences in the study populations. Hakim et al excluded women with anovulatory cycles, and Jensen et al included only nulliparous women. By contrast, we included all women trying to conceive. However, we stratified the analysis on parity and found that the inverse association did not vary consistently by parity, and as the numbers of participants in these subgroups were small, the observed variation may reasonably be explained by chance.

The other Danish study, based on retrospective ascertainment of alcohol and time to pregnancy among 29 844 pregnant women, found that wine drinkers at any level of consumption conceived more quickly than non-wine drinkers.^16^ Our study and that of Jensen et al did not corroborate this finding.^11^

The biological mechanisms by which alcohol could impair fertility are complex and poorly understood. Excessive alcohol consumption may adversely affect fecundability through alterations in endogenous hormones.^40^ ^41^ In a crossover study of 34 premenopausal women, Reichman et al found that an intake of 14 drinks a week was associated with increased concentrations of total estrogen and amount of bioavailable estrogen, compared with no intake.^42^ Similarly, in another study of 790 premenopausal women, consumption of more than 25 g of alcohol per day (approximately 14 drinks a week) was associated with higher levels of sex hormones, compared with no intake.^43^ In a cross sectional study of 498 non-pregnant women, Lucero et al found that the mean concentration of estradiol (E_2_) was 3.42 pg/mL for women consuming less than one drink a day compared with 3.60 pg/mL for women consuming one or more drinks a day.^44^ Thus, consumption of high amounts of alcohol may affect endogenous hormone concentrations in a manner that reduces fecundability.

### Conclusion

In summary, our study showed that consumption of 14 or more servings of alcohol a week was slightly associated with reduced fecundability, but consumption of lower amounts seemed to have no discernible effect on fertility. Nonetheless, because the fetus may be particularly vulnerable to alcohol during the first few weeks after conception, it would seem prudent for women who are actively trying to become pregnant to abstain from alcohol during their fertile window until a pregnancy has been ruled out.

**What is already know on this topic**

Women trying to become pregnant are advised to abstain from alcohol consumption, although the extent to which alcohol consumption affects female fecundity is unclear
Some studies have reported that low to moderate levels of alcohol consumption are associated with decreased fertility
Other studies reported no association or even a positive association between moderate alcohol intake and fertility

**What this study adds**

Consumption of less than 14 servings of alcohol a week seemed to have no discernible effect on fertility
No appreciable difference in fecundability by level of consumption of beer and wine was apparent

## Notes

We are grateful to Tina Christensen for her support in data collection and media contacts. We thank all the women who participated.

Ethical approval: The study was approved by the Danish Data Protection Agency (J.nr. 2013-41-1922) and the Institutional Review Board at Boston University. Ethical approval was not needed according to the Danish Ethical Review System.

Data sharing: No additional data available.

Transparency: The lead author affirms that the manuscript is an honest, accurate, and transparent account of the study being reported; that no important aspects of the study have been omitted; and that any discrepancies from the study as planned (and, if relevant, registered) have been explained.
